# Spaceflight Promotes Biofilm Formation by *Pseudomonas aeruginosa*

**DOI:** 10.1371/journal.pone.0062437

**Published:** 2013-04-29

**Authors:** Wooseong Kim, Farah K. Tengra, Zachary Young, Jasmine Shong, Nicholas Marchand, Hon Kit Chan, Ravindra C. Pangule, Macarena Parra, Jonathan S. Dordick, Joel L. Plawsky, Cynthia H. Collins

**Affiliations:** 1 Department of Chemical & Biological Engineering, Rensselaer Polytechnic Institute, Troy, New York, United States of America; 2 Center for Biotechnology and Interdisciplinary Studies, Rensselaer Polytechnic Institute, Troy, New York, United States of America; 3 Lockheed Martin, Ames Research Center, Moffett Field, California, United States of America; Institut Pasteur, URA CNRS 2172, France

## Abstract

Understanding the effects of spaceflight on microbial communities is crucial for the success of long-term, manned space missions. Surface-associated bacterial communities, known as biofilms, were abundant on the Mir space station and continue to be a challenge on the International Space Station. The health and safety hazards linked to the development of biofilms are of particular concern due to the suppression of immune function observed during spaceflight. While planktonic cultures of microbes have indicated that spaceflight can lead to increases in growth and virulence, the effects of spaceflight on biofilm development and physiology remain unclear. To address this issue, *Pseudomonas aeruginosa* was cultured during two Space Shuttle Atlantis missions: STS-132 and STS-135, and the biofilms formed during spaceflight were characterized. Spaceflight was observed to increase the number of viable cells, biofilm biomass, and thickness relative to normal gravity controls. Moreover, the biofilms formed during spaceflight exhibited a column-and-canopy structure that has not been observed on Earth. The increase in the amount of biofilms and the formation of the novel architecture during spaceflight were observed to be independent of carbon source and phosphate concentrations in the media. However, flagella-driven motility was shown to be essential for the formation of this biofilm architecture during spaceflight. These findings represent the first evidence that spaceflight affects community-level behaviors of bacteria and highlight the importance of understanding how both harmful and beneficial human-microbe interactions may be altered during spaceflight.

## Introduction

Manned space missions conducted over the past 50 years have expanded our knowledge of the universe, and have led to the identification of a range of challenges that must be addressed as we move towards the next phase of human space exploration. A key concern is how microgravity and other aspects of the spaceflight environment affect bacterial growth, physiology and virulence. Several in-flight studies have reported that the microgravity environment encountered during spaceflight can alter bacterial growth and physiology, including increased final cell density, antibiotic resistance, and virulence (reviewed by Horneck *et al.* [1]). However, the effects of spaceflight on microbial community behaviors, such as biofilm formation, have not been systematically addressed. Furthermore, spaceflight has been shown to have harmful effects on astronauts including decreased immune system function [2]. Recent in-flight studies using *Drosophila* and mouse models have also shown that spaceflight can suppress the innate immune system [3], [4]. The combined microgravitational effects of decreased immune function in space travelers and increased resistance and virulence in bacteria may be detrimental to the health of the crew during long-term space exploration.

To date, spaceflight studies on bacterial behavior have been conducted using suspension cultures. However, such cultures do not typically represent real-world situations because the majority of bacteria in nature exist in surface-associated microbial communities known as biofilms. Bacteria in biofilms often exhibit increased resistance to environmental stress, antibiotics, and host defense systems [5]. Thus, many problems caused by biofilms such as biofouling, corrosion, and infectious diseases are difficult to control using conventional antimicrobial treatments [5], [6]. Indeed, abundant biofilms were found in the Russian Mir space station and were responsible for increased corrosion and a blocked water purification system [7]. Ground-based studies have indicated that corrosion caused by biofilms can be detrimental to materials that were used on the International Space Station and other spacecrafts [8]. An experiment using simulated microgravity showed that *Escherichia coli* grown in simulated microgravity form thicker biofilms and exhibit increased resistance to stress compared to normal gravity controls [9]. In experiments conducted aboard STS-95, McLean *et al.* observed that *Pseudomonas aeruginosa* is able to form biofilms during spaceflight [10]. However, their experimental system did not enable quantitative comparisons of biofilms formed during spaceflight and normal gravity. More recently, Wilson *et al.* observed that *Salmonella* cultured during spaceflight exhibited increases in cellular aggregation and clumping, processes that are associated with biofilm formation [11]. Despite such evidence that biofilm formation may be altered in a low gravity environment, systematic studies comparing biofilm formation during spaceflight and normal gravity have not been reported.

To examine the role of microgravity on microbial biofilm formation, we conducted two sets of experiments aboard the Space Shuttle Atlantis (STS-132 and STS-135) where we examined the growth of *P. aeruginosa* PA14, an opportunistic human pathogen and a model organism for biofilm studies. Previous studies have examined how planktonic *P. aeruginosa* cells respond to simulated microgravity and the spaceflight environment [12]–[16]. Crabbé *et al.,* identified 167 genes and 28 proteins that were differentially regulated during spaceflight, and showed that the global regulator Hfq plays a key role in how *P. aeruginosa* responds to microgravity [16]. To study *P. aeruginosa* biofilm formation during spaceflight, we used specialized hardware designed for growing cells during spaceflight, known as a fluid processing apparatus (FPA; Figure S1). FPAs have been used in several recent studies of bacterial growth and physiology during spaceflight [11], [16], [17]. Briefly, an FPA is a glass barrel that can be divided into compartments by rubber stoppers. During spaceflight, a plunging motion can be used to mix the components loaded into the different compartments via a bevel on the side of the glass barrel. For our experiments, a mixed cellulose ester membrane disc was used as a biofilm substrate. A modified artificial urine media (mAUM, Table S1) was loaded into the first compartment containing the membrane. mAUM was used because it provides a physiologically relevant environment for the study of biofilms formed both inside and outside the human body [18]. The second compartment was filled with inoculum stored in phosphate buffered saline (PBS). For microscopy samples only, a third compartment was filled with a paraformaldehyde solution. As illustrated in the timeline shown in Figure S2, biofilms were formed under static conditions in FPAs at 37°C for 72 h. Subsequently, the temperature was decreased to 8°C to minimize further growth, and fixative was added to the microscopy samples. Samples were obtained approximately 6 h after landing and processed immediately. Ground controls were conducted at Kennedy Space Center in parallel with spaceflight samples.

Here, we report the first evidence that spaceflight affects biofilm formation by *P. aeruginosa*, with increased numbers of viable cells, increased biomass, and increased thickness observed in spaceflight biofilms compared to normal gravity controls. Biofilms formed by *P. aeruginosa* during spaceflight also exhibited a column-and-canopy-shaped architecture that has not been observed previously. We show that flagella-driven motility plays a key role in formation of this novel architecture, and relate the mechanism to structured biofilm formation on Earth.

## Materials and Methods

### Bacterial Strains and Culture Media

Strains used in this study are shown in Table S2. Overnight shaking cultures of all strains were cultured at 37°C in nutrient broth (NB) (Difco BD). To prepare inocula, cultures were washed and resuspended in PBS to a final concentration of ∼6×10^6^ CFU/mL. Modified artificial urine media (mAUM) was used as the growth media (Table S1) [18]. To ensure reproducibility in media composition, RPMI 1640 amino acid solution (50×; Sigma, MO, cat #: R7131,) and L-glutamine solution (200 mM; Sigma, MO, cat #: G7513) were substituted for peptone and yeast extract. The concentration of calcium chloride dihydrate was lowered from 2.5 mM to 0.25 mM to minimize precipitation during storage at 8°C. Sodium nitrate, 6 mM, was added to serve as a terminal electron acceptor and enable cell growth when the conditions in the FPAs become anaerobic. Higher concentrations of sodium nitrate were avoided to minimize any effects on aerobic growth and limit the accumulation of nitrite, which can inhibit cell growth. Phosphate concentration was lowered to 5 mM to decrease phosphate availability. mAUM includes 2 mM citric acid and 5 mM phosphate. To test the effects of phosphate availability on biofilms, the phosphate concentration was increased to 50 mM (mAUM-high Pi). To test the effects of carbon source, 2 mM citric acid was substituted by 2 mM glucose (mAUMg). The pH of all media was adjusted to 7.0.

### Spaceflight and Ground Control Cultures

Spaceflight and ground control cultures were grown in specialized hardware, known as a fluid processing apparatus (FPA), designed by Bioserve Space Technologies (University of Colorado). FPAs consist of a glass barrel, rubber stoppers, and a solid or gas exchange (GE) insert (Figure S1). Glass barrels and rubber stoppers were lubricated by coating with Sigmacote (Sigma, MO) and autoclaved before use. First, a 13 mm diameter Millipore mixed cellulose ester membrane disc (catalog #: GSWP01300) was attached to a solid or GE insert with 3 M autoclavable double-sided tape. Second, a solid or GE insert was inserted to the edge of the bevel in the glass barrel. Next, 2.5 mL of media and 0.5 mL of inoculum were added to the FPA and separated by a rubber stopper. A third compartment, for microscopy samples only, was filled with 2.4 mL of 9% paraformaldehyde. Three biological replicates were prepared for each experimental condition.

To facilitate group activation during spaceflight, eight FPAs were loaded into a single group activation pack (GAP) that enables the simultaneous plunging of rubber stoppers to mix contents of the compartments in all eight FPAs. GAPs were stored in a Commercial Generic Bioprocessing Apparatus (CGBA) that accommodates 16 GAPs and enables temperature control. Cultures were grown following the experiment timeline (Figure S2). Three or five days before shuttle launch, all FPAs and GAPs were loaded. Samples were stored at 8°C. Four or five days before shuttle landing, the media and inoculum were combined and subsequently incubated at 37°C for 72 h. One or two days before shuttle landing, the temperature was decreased to 8°C and the microscopy samples were fixed with paraformaldehyde. An astronaut aboard the shuttle manually changed the incubator temperature and conducted the activation and termination mixing steps. Samples were obtained approximately 6 h after shuttle landing and were processed immediately. Ground controls were conducted at Kennedy Space Center in parallel with spaceflight samples.

### Viable Cell Counting

Mixed cellulose ester membranes were detached from the insert and washed gently three-times with PBS to remove planktonic cells and loosely associated cells. The membrane was placed in 1 mL PBS and sonicated in an ultrasonic bath (Branson 2510) for 8 min. The sonicated samples were serially diluted with PBS in 96-well plate. The diluted samples were spot-plated on NB agar and incubated at 37°C for 18 h.

### Preparation of Biofilm Samples and Microscopic Observation

Mixed cellulose ester membranes were detached and washed as described for viable cell counting. Biofilms were stained with a solution containing 1 µg/mL propidium iodide, 1% Triton X-100 in PBS. The staining procedure was adapted from Castaneda *et al.* [19], where the propidium iodide dye was substituted for fluorescein isothiocyanate (FITC). The use of a 1 µg/mL propidium iodide solution to stain fixed *P. aeruginosa* biofilms has been described previously [20]. Biofilm images were obtained using a Zeiss LSM 510 (Carl Zeiss, Germany) confocal laser scanning microscope equipped with detectors and filter sets for propidium iodide (excitation, 543 nm: emission, 565 nm). Images were obtained using a 40×/1.3 oil objective. Five image stacks (center and 1 mm right, left, up, and down from center) were obtained from each membrane. Simulated three-dimensional images and sections were generated using IMARIS 7.1 software (Bitplane AG, Switzerland). Analysis of confocal images was performed using COMSTAT software [21]. Void fraction was calculated using the following equation:
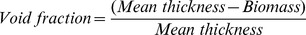



## Results

### Spaceflight Increases Biofilm Formation

**Figure 1:**
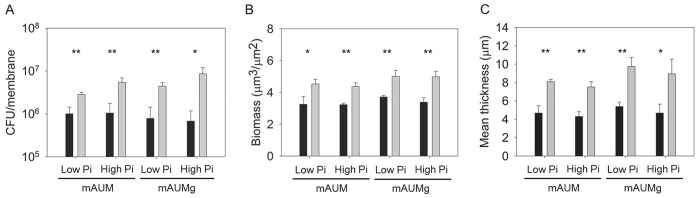
Spaceflight increases biofilm formation by *P. aeruginosa*. Wild-type *P. aeruginosa* was cultured under normal gravity (black bars) and spaceflight (grey bars) conditions in mAUM or mAUMg containing 5 or 50 mM phosphate. (**A**) The number of surface-associated viable cells per cellulose ester membrane. (**B**) Biofilm biomass and (**C**) mean biofilm thickness were quantified by analysis of CLSM images. Error bars, SD; N = 3. *p≤0.05, **p≤*0.01*.

To assess the effects of spaceflight on biofilm formation, we cultured *P. aeruginosa* biofilms and quantitatively compared biofilm formation between spaceflight and normal gravity. The number of viable cells within the biofilms was determined by plate counting. In addition, the biomass (µm^3^ biofilm/µm^2^ membrane) and mean thickness (µm) of the biofilms were determined from images obtained from confocal laser scanning microscopy (CLSM) with the aid of COMSTAT software [21]. The number of viable cells in *P. aeruginosa* biofilms formed in mAUM during spaceflight increased three-fold compared to those formed in normal gravity (*p<0.01*) (Figure 1A). Results from quantitative image analysis also indicated that spaceflight promotes biofilm production by *P. aeruginosa*, where both biomass (*p<0.05*) and mean thickness (*p<0.01*) increased significantly (Figures 1B and 1C).

We tested the role of phosphate availability and carbon source on biofilm formation during spaceflight because previous reports have shown that spaceflight-dependent responses in planktonic cultures of *Salmonella* were modulated by nutrient availability [22]. When the concentration of phosphate in mAUM was increased from 5 mM to 50 mM (mAUM-high Pi), biofilms cultured during spaceflight showed a 5-fold increase in viable cells (*p<0.01*), increased biomass volume (*p<0.01*), and increased mean thickness (*p<0.01*) (Figures 1A–C and Table S4). To assess whether the effect of spaceflight on biofilm formation is dependent on carbon source, we substituted 2 mM citric acid in mAUM with 2 mM glucose (mAUMg). As with mAUM, *P. aeruginosa* biofilms cultured in mAUMg and mAUMg-high Pi during spaceflight exhibited a 6- to 12-fold increase in viable cell counts (*p<0.01*), along with corresponding increases in biomass (*p<0.01*) and mean thickness (*p<0.01*), compared to normal gravity controls (Figure 1, Tables 1 and S4). Overall, we have observed that spaceflight induced an increase in the number of viable cells, biomass, and mean thickness of biofilms regardless of phosphate concentration or carbon source. The experiments with mAUM and mAUM-high Pi were conducted both on STS-132 and aboard STS-135. The results from STS-135 (Figure 1) were consistent with those from STS-132 (Figure S3). Planktonic growth was also observed to increase during spaceflight only under the low phosphate conditions found in mAUM and mAUMg (manuscript in preparation). However, no differences between spaceflight and normal gravity planktonic cell growth were observed when the concentration of phosphate in the media was increased to 50 mM, indicating that the observed increases in biofilm biomass are not solely due to increases in bacterial growth.

**Table 1:** Spaceflight and motility affect biofilm formation and architecture.

*P. aeruginosa*	Gravity	Viable cells(10^6^ CFU/membrane)	Biomass(µm^3^/µm^2^)	Mean thickness(µm)	Void fraction	Structure
Wild type	Normal gravity	0.8±0.6	3.7±0.1	5.4±0.5	0.26±0.05	Flat
	Spaceflight	4.4±0.9	5.0±0.4	9.8±1.0	0.47±0.02	Column & canopy
*ΔmotABCD*	Normal gravity	0.3±0.1	3.9±0.3	5.7±0.1	0.31±0.06	Flat
	Spaceflight	3.1±1.4	4.2±0.4	6.1±0.5	0.30±0.01	Flat
*ΔpilB*	Normal gravity	2.1±1.0	3.9±0.3	5.3±0.2	0.25±0.05	Flat
	Spaceflight	5.6±1.1	4.9±0.4	9.2±1.1	0.47±0.04	Column & canopy
Wild type (GE)	Normal gravity	ND	6.4±0.2	8.7±0.7	0.25±0.04	Flat
	Spaceflight	ND	6.2±0.1	8.7±0.3	0.28±0.02	Flat
*ΔmotABCD* (GE)	Normal gravity	ND	4.1±0.3	5.6±0.5	0.26±0.03	Flat
	Spaceflight	ND	4.1±0.5	5.8±0.9	0.28±0.06	Flat

Wild type, *ΔmotABCD,* and *ΔpilB* were grown in mAUMg with solid inserts or GE inserts. Biomass and mean thickness were calculated from CLSM images using COMSTAT. Results are shown as mean ± SD; N = 3. ND, not determined.

### Spaceflight Alters Biofilm Architecture

**Figure 2:**
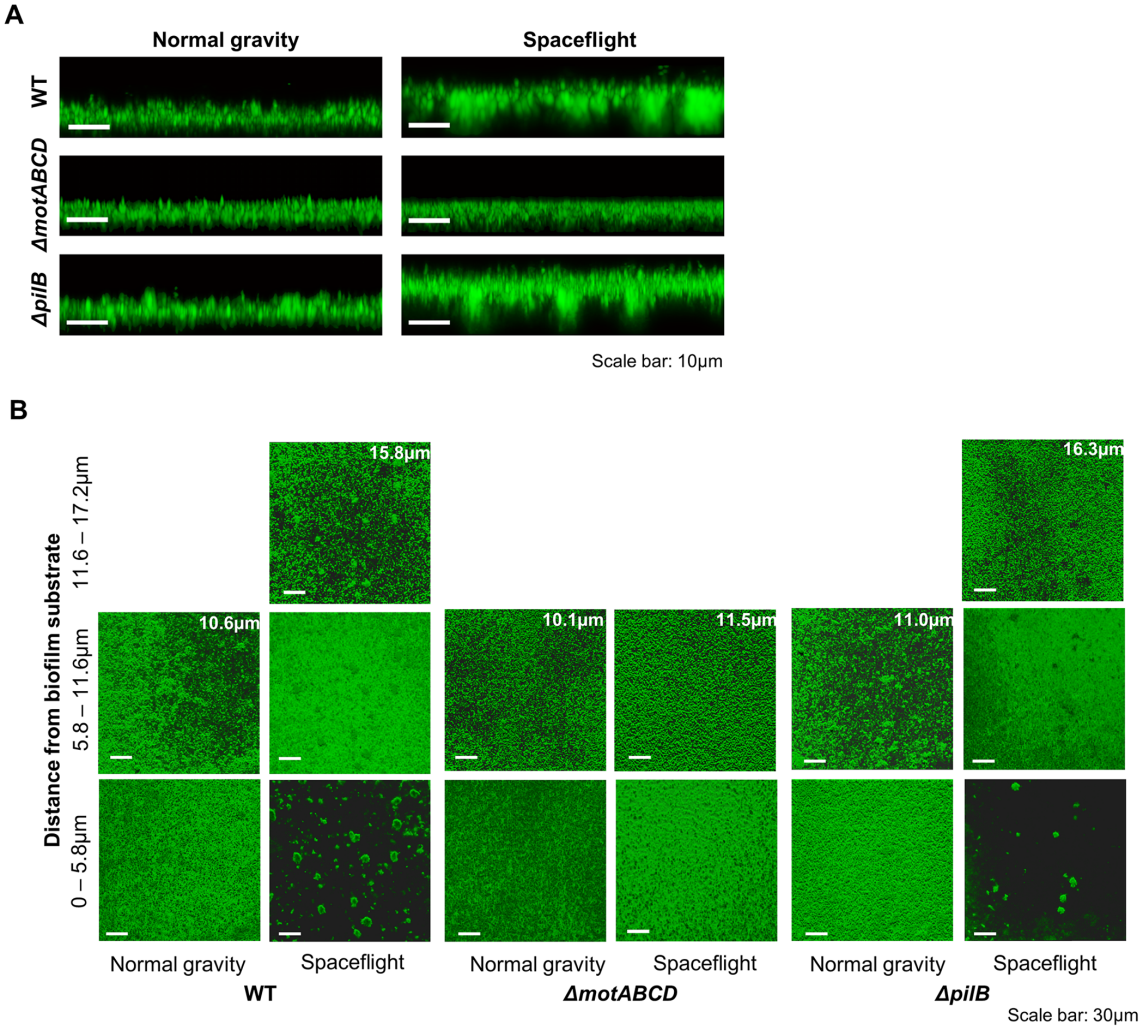
*P.aeruginosa* biofilms cultured during spaceflight display column-and-canopy structures. Confocal laser scanning micrographs of 3-day-old biofilms formed by wild type, *ΔmotABCD*, and *ΔpilB* comparing normal gravity and spaceflight culture conditions. All strains were grown in mAUMg with 5 mM phosphate. No significant differences in structure or thickness were observed with mAUMg containing 5 or 50 mM phosphate. (**A**) Representative side-view images. (**B**) Representative 5.8 µm thick slices generated from partial *z* stacks. Maximum thickness is indicated in the upper right corner of the top slice for each condition.

On Earth, *P. aeruginosa* forms mushroom-shaped, structured biofilms under hydrodynamic conditions, such as those found in flow-cell systems, in media containing glucose as a carbon source and flat biofilms in media with citrate [23]–[25]. Under static conditions, structured biofilms are generally not observed because of limited nutrient availability and aeration [5], [26]. To assess whether spaceflight causes any structural differences in *P. aeruginosa* biofilms, we compared CLSM images obtained from samples grown in spaceflight and normal gravity. The increased thickness of the biofilms formed in mAUMg during spaceflight is readily apparent from side view images (Figure 2A). Moreover, *P. aeruginosa* biofilms grown during spaceflight exhibited a structure consisting of columns overlaid with a canopy, while biofilms cultured in normal gravity showed flat structures. As shown in Figure 2B, the column-and-canopy structure can be seen clearly when slices of the biofilm, approximately 5.8 µm thick, were prepared from partial *z* stacks. The slice closest to the substratum clearly shows cell aggregates that have formed column-like structures with significant unoccupied space. Farther away from the membrane, biofilms grown in spaceflight showed dense, mat-like structures that form a canopy over the columns. In contrast, the normal gravity samples showed uniformly dense structures. Comparisons of the *z* stack slices showed that *P. aeruginosa* grown during spaceflight formed column-and-canopy structured biofilms in mAUM (Figure S4), as well as mAUM-high Pi and mAUMg-high Pi (Table S4). To the best of our knowledge, such structures have not been reported previously. Further, the presence of three-dimensionally structured, as opposed to flat, biofilms was especially surprising due to the static environment in our biofilm culture system.

Quantitative image analysis comparing the amount of unoccupied space within the biofilm structure also indicated a significant structural difference between biofilms formed in normal gravity and those formed during spaceflight. Specifically, we compared the void fraction of biofilms using the biomass and mean thickness values obtained from CLSM image analysis. Biofilms formed during spaceflight exhibited a 1.8-fold increase in void fraction compared to normal gravity controls (Tables 1 and S3). This observation is consistent with the large volume of empty space observed “underneath” the biofilm canopies formed under microgravity.

### Motility Affects Spaceflight Biofilm Formation

Flagella-driven motility and type IV pili-driven motility have been shown to affect *P. aeruginosa* biofilm development [25], [27]. Furthermore, flagella-driven motility plays a key role in the development of structured biofilms under hydrodynamic conditions [28]. To examine whether motility plays a role in the formation of the column-and-canopy-shaped biofilms during spaceflight we compared CLSM images obtained with wild-type *P. aeruginosa* with those formed by mutants deficient in flagella-driven motility, *ΔmotABCD* [29], and type IV pili-driven motility, *ΔpilB* [27]. As shown in Figure 2, the structure of *ΔmotABCD* biofilms cultured during spaceflight showed uniformly dense biofilms with no apparent structural difference from those cultured in normal gravity (Figures 2 and S4). In contrast, *ΔpilB* behaved similarly to wild type, where biofilms formed during spaceflight showed column-and-canopy structures and those formed in normal gravity showed dense, uniform biofilms (Figure 2). These findings indicate that, like the mushroom-shaped structured biofilms formed on Earth, flagella-driven motility plays a key role in formation of column-and-canopy structured biofilms.

We also assessed the role of motility on biofilm production. Like wild-type *P. aeruginosa*, *ΔmotABCD* grown during spaceflight showed an 8-fold increase in number of viable cells in biofilms compared to those grown in normal gravity regardless of carbon source (Tables 1 and S3). From COMSTAT image analysis, however, no significant difference in biomass or mean thickness was observed in *ΔmotABCD* biofilms cultured in mAUM or mAUMg between spaceflight and normal gravity (Tables 1 and S3). The discrepancy between viable cell counting and COMSTAT analysis in *ΔmotABCD* biofilms indicates a difference in relative numbers of viable cells per volume of biomass, where spaceflight may increase either the amount of cells per volume of extracellular matrix or the viability of cells within the matrix. Like wild-type *P. aeruginosa*, *ΔpilB* biofilms grown during spaceflight showed increased viable cell numbers in biofilms (*p<0.01*), biomass (*p<0.05*), and mean thickness (*p<0.01*) compared to normal gravity controls (Table 1).

### Effects of Oxygen Availability on Biofilm Formation

**Figure 3:**
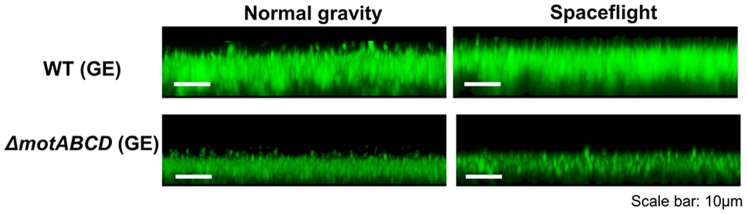
Increased oxygen availability minimizes gravitational effects on biofilm formation by *P.aeruginosa*. Representative side view confocal laser scanning micrographs of 3-day-old biofilms formed by wild-type *P. aeruginosa* and *ΔmotABCD* grown in mAUMg with gas exchange (GE) inserts comparing normal gravity and spaceflight culture conditions.

To assess the effect of oxygen availability on biofilm formation during spaceflight, we substituted the solid inserts used in the experiments described above with gas exchange (GE) inserts that allow the movement of gases via a gas permeable membrane (Figure S1). A significant increase in biofilm formation was observed with GE inserts compared to solid inserts under both normal gravity and microgravity conditions (Figure 3 and Table 1). Further, no differences between biofilms grown under normal and microgravity conditions were observed with the GE inserts for both wild type and *ΔmotABCD*. The structures of biofilms grown with GE inserts were flat and dense (Figure 3 and Table 1), similar to those observed under static conditions on Earth [26]. We also observed that the difference between the amount of planktonic biomass formed in normal gravity and spaceflight was minimized with the GE inserts (manuscript in preparation).

## Discussion

**Figure 4:**
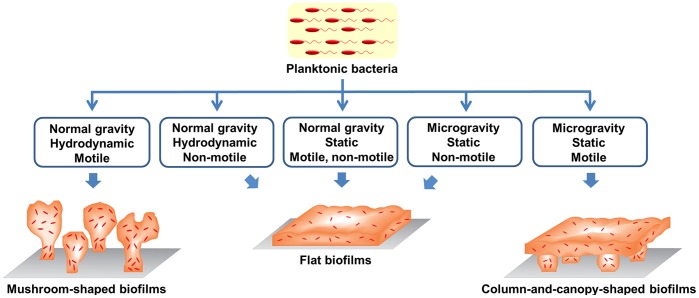
Illustration summarizing the influence of gravity, flow, and motility on *P.aeruginosa* biofilm architecture.

We have shown that *P. aeruginosa* forms column-and-canopy-shaped biofilms during spaceflight and that flagella-driven motility plays a key role in the formation of this unique structure. Figure 4 summarizes how biofilms formed under the spaceflight culture conditions compare with those formed under two common laboratory culture conditions, static and hydrodynamic [26], [30]. Under hydrodynamic conditions, *P. aeruginosa* can form mushroom-shaped structured biofilms, while flat biofilms are generally observed under static conditions [23]. Under static conditions during spaceflight, however, biofilms with column-and-canopy structures were observed. Flagella-driven motility plays a key role the formation of column-and-canopy-shaped biofilms formed during spaceflight and the mushroom-shaped biofilms formed in hydrodynamic conditions on Earth. However, the formation of mushroom-shaped biofilms is dependent on a carbon source [31], while we have observed that the formation of column-and-canopy-shaped biofilms is independent of carbon source (Figures 2 and S4). Both the column-and-canopy-shaped biofilms and the mushroom-shaped biofilms have two-layered structures, and the mushroom “stalks" and columns are likely formed using the same mechanism, further evidenced by the fact that flagella-driven motility is required for both. While the top layer of the mushroom-shaped biofilms is an isolated cap structure, the top layer of the column-and-canopy-shaped biofilms is a flat mat-like structure. The canopy is likely an extension and connection of the isolated “caps” observed in mushroom-shaped structures (Figure 4). While still an active area of research, the size of mushroom “cap” has been observed to be limited under hydrodynamic conditions in normal gravity by a combination of flow [32], gravity or natural dispersal mechanisms [5]. In contrast, the fluid dynamics surrounding cells in microgravity are extremely quiescent because of diminished gravity (∼10^−6^ g) [33], [34]. Under microgravity conditions, the virtual absence of flow and gravity may promote the unlimited expansion of the mushroom “caps” resulting in the formation of the intact canopy on top of the relatively widely spaced columns. The formation of flat biofilms with the GE inserts may be due an increase in biofilm growth rate, either by preventing the formation of the stratified structure or expanding into the space available under the canopy. However, it is possible that increased oxygen availability inhibits the formation of the column-and-canopy structure.

We speculate that the increases in biofilm formation observed with the GE inserts are primarily due to increased oxygen availability. With the solid inserts, the conditions in the FPA are most likely microaerophilic to anaerobic as the cells consume the oxygen available in the liquid and air bubble present in the closed system. While the GE inserts were designed to provide an aerobic environment in the FPAs, results from *P. aeruginosa* gene expression studies have indicated that growth is likely oxygen limited even with the GE inserts and that differences in oxygen gradients within the FPAs likely exist between normal gravity and spaceflight conditions [16]. In the present study, the direct attachment of the biofilm substrate to the GE inserts maximized the oxygen available for surface-associated cells and minimized the differences in oxygen availability between spaceflight and normal gravity conditions. Overall, in the samples grown with the solid inserts, oxygen is likely a limiting substrate for growth. When the GE inserts were used to increase the oxygen availability, an increase in biofilm was observed and the difference between normal gravity and microgravity diminished. Therefore, the advantage conferred by microgravity is dependent upon resource availability, where changes in mixing, diffusion, sedimentation and other environmental factors play a more significant role under such limiting conditions. This observation is similar to results from Wilson *et al.*, who showed that an increase in nutrient availability minimized the differences in virulence observed with *Salmonella* between normal and microgravity [22]. We anticipate that these data will lead to the development of mathematical models describing the physico-chemical environment experienced by the cells under microgravity and normal gravity conditions and insights into the nature of the differences that cause the observed changes in growth and dependence on nutrient availability.

We have demonstrated that spaceflight can trigger changes in *P. aeruginosa* biofilm growth and architecture. To the best of our knowledge, these findings are the first evidence that spaceflight influences community-level behaviors of bacteria. Our findings indicate that altered biofilm formation during spaceflight may have detrimental impacts on long-term spaceflight missions, where increases in biofouling and microbially-induced corrosion could have profound impacts on mission success. Furthermore, it will be important to explore the effects of such changes on human health through pathogenic and beneficial interactions between humans and microbes during spaceflight.

While further studies are required to elucidate the mechanisms involved in the formation of the unique biofilm architecture observed during spaceflight, this work provides a set of data exploring conditions and parameters that are not possible on Earth. Thus, furthering our understanding of how environmental parameters alter biofilm formation by this key opportunistic pathogen. The unique morphology of the *P. aeruginosa* biofilms formed in microgravity suggests that nature is capable of adapting to non-terrestrial environments in ways that deserve further studies, including those exploring long-term growth and adaptation to a low gravity environment.

## Supporting Information

Figure S1 **Specialized hardware for spaceflight experiments. (A)** Fluid processing apparatus (FPA) loaded with colored water to illustrate experimental setup. A mixed cellulose membrane was attached with two-sided tape to either a solid insert or a gas exchange insert. 2.5 mL of media was loaded into the first compartment (blue). 0.5 mL of inoculum was loaded into the second compartment (yellow). For microscopy samples only, 2.4 mL of a 9% (w/v) solution of paraformaldehyde in PBS was loaded into the compartment (red). **(B)** Group activation pack (GAP). A representative GAP loaded with samples for viable cell counting. Mixing of the contents is achieved by use of a crank handle attached to the top of the GAP, enabling uniform plunging of each FPA. **(C)** Commercial generic bioprocessing apparatus (CGBA). The CGBA functions as an incubator and holds 16 GAPs. The CGBA functions as an incubator and holds 16 GAPs. The CGBA, containing 16 GAPs, was loaded directly into a middeck locker aboard the space shuttle. Temperature changes were made manually by an astronaut during spaceflight.
(PDF)

Figure S2 **Timeline of spaceflight experiments.** Ground controls were conducted at Kennedy Space Center in parallel with spaceflight samples conducted on the Space Shuttle Atlantis.
(PDF)

Figure S3 ***P. aeruginosa*** **biofilms cultured during STS-132 exhibited increased biofilm formation.** Wild-type *P. aeruginosa* was cultured under normal gravity (black bars) and spaceflight (grey bars) conditions in mAUM containing 5 or 50 mM phosphate. **(A)** The number of surface-associated viable cells per cellulose ester membrane. **(B)** Biofilm biomass and **(C)** mean biofilm thickness were quantified by analysis of CLSM images. Error bars, SD; N = 3. *p≤0.05, **p≤*0.01*.
(PDF)

Figure S4 ***P. aeruginosa*** **biofilms cultured in mAUM during spaceflight display column-and-canopy structures.** Confocal laser scanning micrographs of 3-day-old biofilms formed by wild type and *ΔmotABCD* comparing normal gravity and spaceflight culture conditions. No significant differences in structure were observed with mAUM containing 5 or 50 mM phosphate. **(A)** Representative side-view images. **(B)** Representative 5.8 µm thick slices generated from partial *z* stacks. Maximum thickness is indicated in the upper right corner of the top slice for each condition.
(PDF)

Table S1 Composition of modified artificial urine media (mAUM).
(PDF)

Table S2 Bacterial strains.
(PDF)

Table S3 Spaceflight and motility affect biofilm formation and architecture in mAUM.
(PDF)

Table S4 Effects of spaceflight and motility on biofilm formation and architecture in high phosphate media.
(PDF)
