# Supplementary figures and images for: Spaceflight Promotes Biofilm Formation by Pseudomonas aeruginosa
Source: PLoS One. 2013 Apr 29;8(4):e62437. doi: 10.1371/journal.pone.0062437 (PMC3639165; doi:10.1371/journal.pone.0062437)

**A**

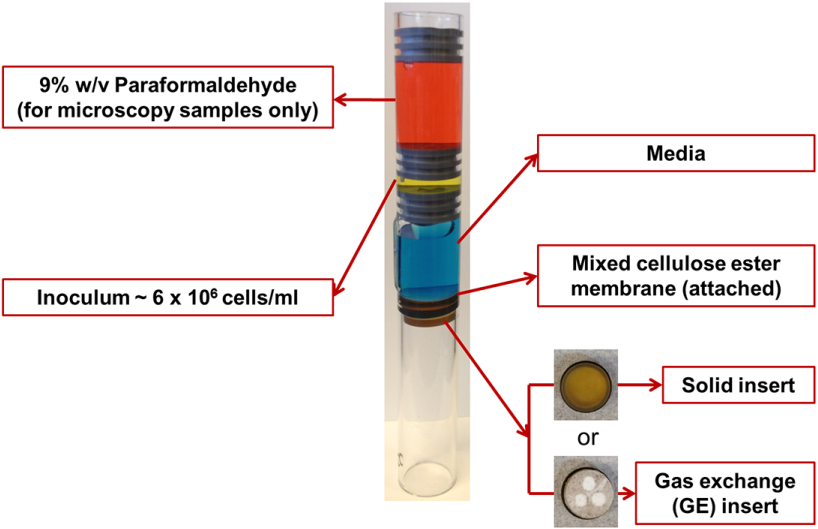

**B**

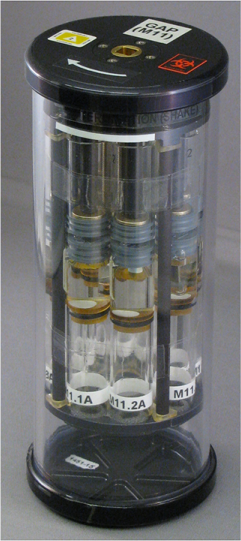

**C**

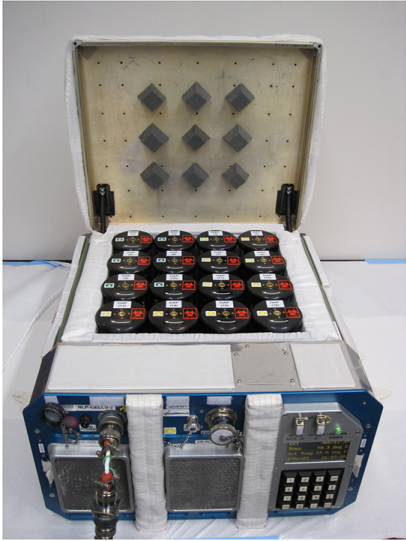

Supplement: Figure S1 — Specialized hardware for spaceflight experiments. (A) Fluid processing apparatus (FPA) loaded with colored water to illustrate experimental setup. A mixed cellulose membrane was attached with two-sided tape to either a solid insert or a gas exchange insert. 2.5 mL of media was loaded into the first compartment (blue). 0.5 mL of inoculum was loaded into the second compartment (yellow). For microscopy samples only, 2.4 mL of a 9% (w/v) solution of paraformaldehyde in PBS was loaded into the compartment (red). (B) Group activation pack (GAP). A representative GAP loaded with samples for viable cell counting. Mixing of the contents is achieved by use of a crank handle attached to the top of the GAP, enabling uniform plunging of each FPA. (C) Commercial generic bioprocessing apparatus (CGBA). The CGBA functions as an incubator and holds 16 GAPs. The CGBA functions as an incubator and holds 16 GAPs. The CGBA, containing 16 GAPs, was loaded directly into a middeck locker aboard the space shuttle. Temperature changes were made manually by an astronaut during spaceflight. (PDF) [file pone.0062437.s001.pdf]

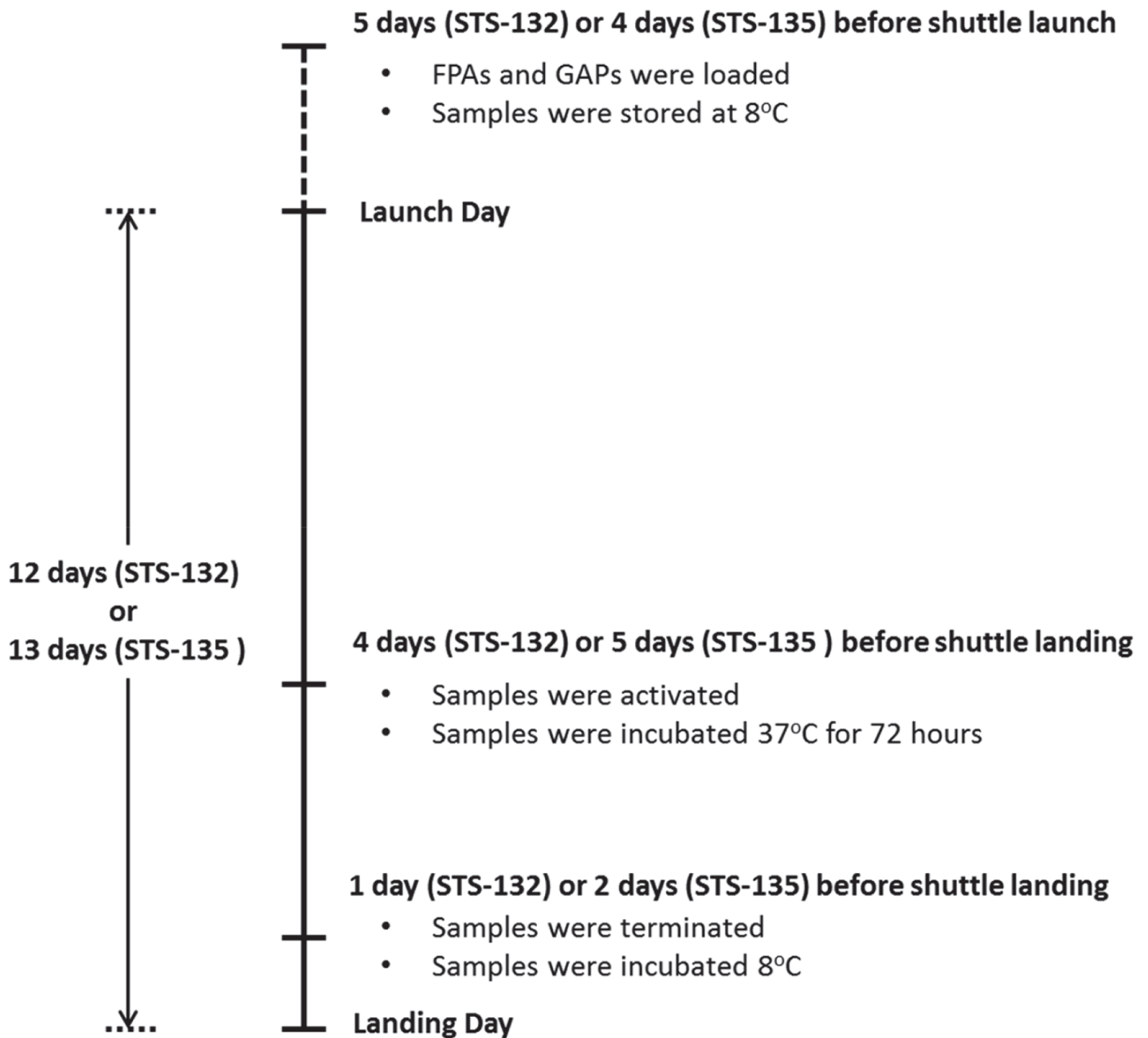

Supplement: Figure S2 — Timeline of spaceflight experiments. Ground controls were conducted at Kennedy Space Center in parallel with spaceflight samples conducted on the Space Shuttle Atlantis. (PDF) [file pone.0062437.s002.pdf]

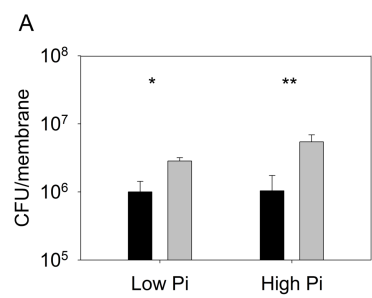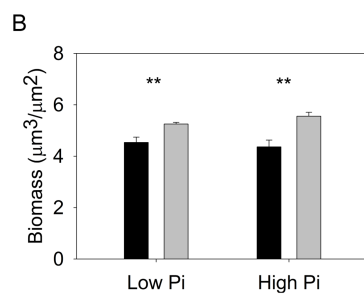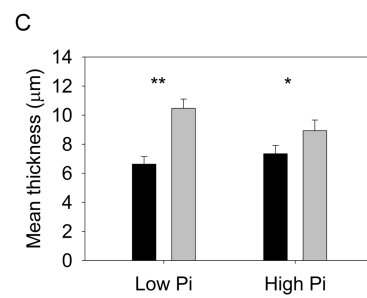

Supplement: Figure S3 — P. aeruginosa biofilms cultured during STS-132 exhibited increased biofilm formation. Wild-type P. aeruginosa was cultured under normal gravity (black bars) and spaceflight (grey bars) conditions in mAUM containing 5 or 50 mM phosphate. (A) The number of surface-associated viable cells per cellulose ester membrane. (B) Biofilm biomass and (C) mean biofilm thickness were quantified by analysis of CLSM images. Error bars, SD; N = 3. *p≤0.05, **p≤0.01. (PDF) [file pone.0062437.s003.pdf]

**A**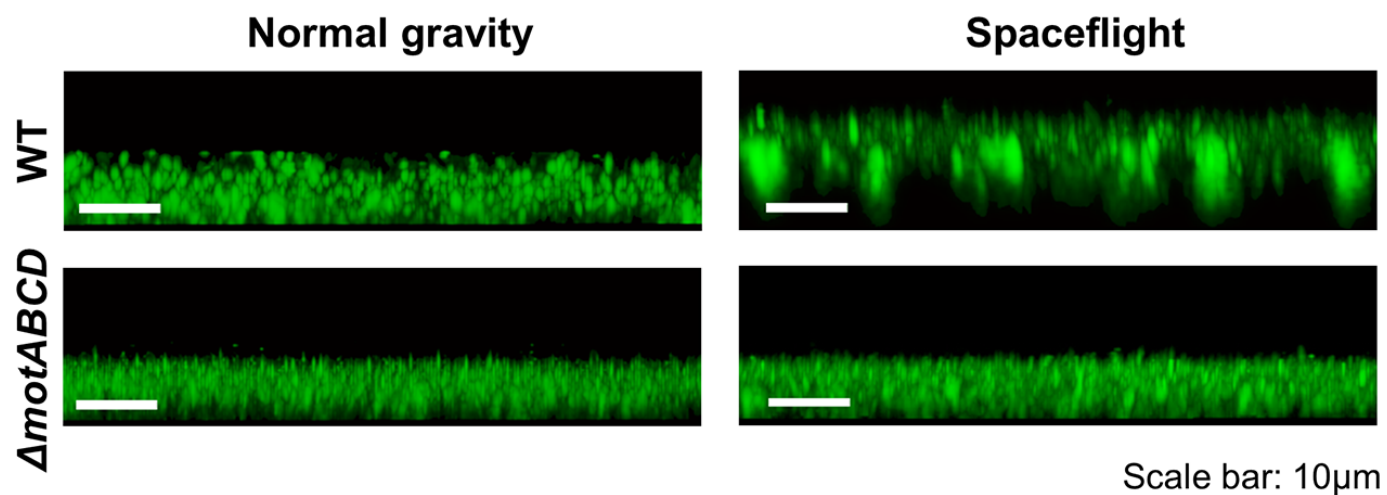**B**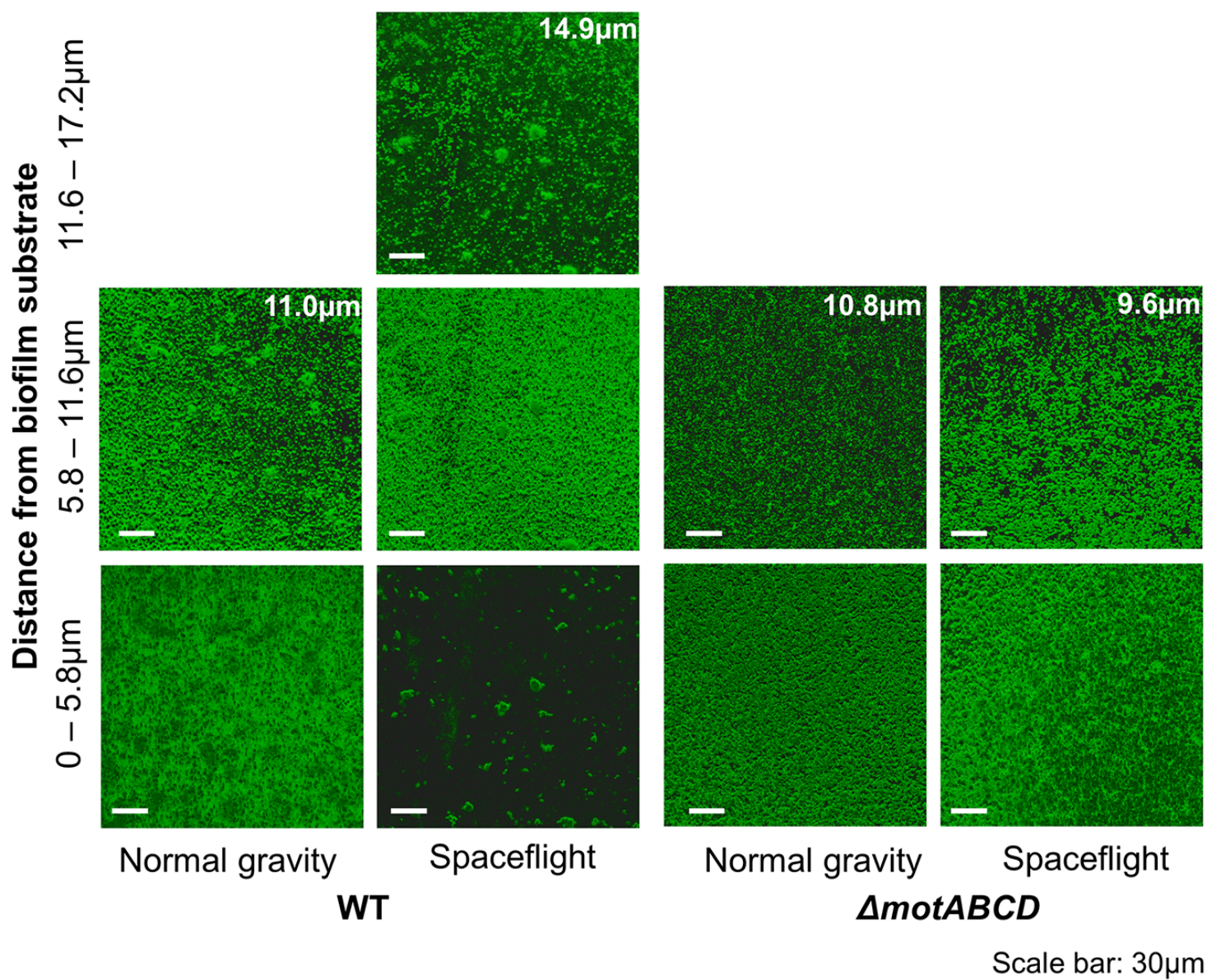

Supplement: Figure S4 — P. aeruginosa biofilms cultured in mAUM during spaceflight display column-and-canopy structures. Confocal laser scanning micrographs of 3-day-old biofilms formed by wild type and ΔmotABCD comparing normal gravity and spaceflight culture conditions. No significant differences in structure were observed with mAUM containing 5 or 50 mM phosphate. (A) Representative side-view images. (B) Representative 5.8 µm thick slices generated from partial z stacks. Maximum thickness is indicated in the upper right corner of the top slice for each condition. (PDF) [file pone.0062437.s004.pdf]
