# Supplementary material for: Spaceflight Promotes Biofilm Formation by Pseudomonas aeruginosa
Source: PLoS One. 2013 Apr 29;8(4):e62437. doi: 10.1371/journal.pone.0062437 (PMC3639165; doi:10.1371/journal.pone.0062437)
Supplement: Table S1 — Composition of modified artificial urine media (mAUM). (PDF) [file pone.0062437.s005.pdf]

Table S1. Composition of modified artificial urine media (mAUM)

Component	mAUM	mAUM-high Pi	mAUMg	mAUMg-high Pi
Citric acid	2 mM	2 mM		
**Glucose**			**2 mM**	**2 mM**
Lactic acid	1.1 mM	1.1 mM	1.1 mM	1.1 mM
Sodium chloride	90 mM	90 mM	90 mM	90 mM
Ammonium chloride	25 mM	25 mM	25 mM	25 mM
**RPMI 1640 amino acid solution (50x; Sigma, MO, cat #: R7131)**	**20 ml/l**	**20 ml/l**	**20 ml/l**	**20 ml/l**
**L-glutamine (200mM; Sigma, MO, cat #: G7513)**	**2 mM**	**2 mM**	**2 mM**	**2 mM**
Urea	170 mM	170 mM	170 mM	170 mM
Uric acid	0.4 mM	0.4 mM	0.4 mM	0.4 mM
Creatinine	7 mM	7 mM	7 mM	7 mM
**Calcium chloride.2H_2O**	**0.25 mM**	**0.25 mM**	**0.25 mM**	**0.25 mM**
Magnesium sulphate.7H_2O	2 mM	2 mM	2 mM	2 mM
Sodium sulphate.10H_2O	10 mM	10 mM	10 mM	10 mM
Sodium bicarbonate	25 mM	25 mM	25 mM	25 mM
**Sodium nitrate**	**6 mM**	**6 mM**	**6 mM**	**6 mM**
Iron II sulphate solution	0.005 mM	0.005 mM	0.005 mM	0.005 mM
**Potassium dihydrogen phosphate**	**1.8 mM**	**28.8 mM**	**1.8 mM**	**28.8 mM**
**Di-potassium hydrogen phosphate**	**1.8 mM**	**28.8 mM**	**1.8 mM**	**28.8 mM**

An artificial urine medium described by Brooks *et al.* [1] was adapted for these experiments (changes are highlighted in bold). The final concentration of phosphate in mAUM, mAUMg and mAUM-high Pi, mAUMg-high Pi was 5 mM and 50 mM, respectively when combined with the PBS-containing inocula. pH adjusted to 7.

1. Brooks T, Keevil CW (1997) A simple artificial urine for the growth of urinary pathogens. Lett Appl Microbiol 24: 203-206.
