# Supplementary material for: Spaceflight Promotes Biofilm Formation by Pseudomonas aeruginosa
Source: PLoS One. 2013 Apr 29;8(4):e62437. doi: 10.1371/journal.pone.0062437 (PMC3639165; doi:10.1371/journal.pone.0062437)
Supplement: Table S2 — Bacterial strains. (PDF) [file pone.0062437.s006.pdf]

Table S2. Bacterial strains

Strain	Relevant characteristics	Reference
WT	*P. aeruginsoa* PA14. Wild type.	[1]
ΔmotABCD	Flagella-driven motility deficient mutant. *motAB* and *motCD* were inactivated in *P. aeruginosa* PA14 by allelic displacement with a gentamycin-resistance cassette using pEX18-*motAB* and pEX18-*motCD*	[2]
ΔpilB	Type IV pili-dependent motility deficient mutant. *pilB* inactivated in *P. aeruginsoa* PA14 by the transposon Tn5-B30(Tc^r)	[3]

1. Rahme LG, Stevens EJ, Wolfort SF, Shao J, Tompkins RG, et al. (1995) Common virulence factors for bacterial pathogenicity in plants and animals. Science 268: 1899-1902.
2. Toutain CM, Zegans ME, O'Toole GA (2005) Evidence for two flagellar stators and their role in the motility of *Pseudomonas aeruginosa*. J Bacteriol 187: 771-777.
3. O'Toole GA, Kolter R (1998) Flagellar and twitching motility are necessary for *Pseudomonas aeruginosa* biofilm development. Mol Microbiol 30: 295-304.
