# Supplementary material for: Spaceflight Promotes Biofilm Formation by Pseudomonas aeruginosa
Source: PLoS One. 2013 Apr 29;8(4):e62437. doi: 10.1371/journal.pone.0062437 (PMC3639165; doi:10.1371/journal.pone.0062437)
Supplement: Table S3 — Spaceflight and motility affect biofilm formation and architecture in mAUM. (PDF) [file pone.0062437.s007.pdf]

Table S3. Spaceflight and motility affect biofilm formation and architecture in mAUM.

P. aeruginosa	Gravity	Viable cells (10^6 CFU/mem)	Biomass ($\mu m^3/\mu m^2$)	Mean thickness (μm)	Void fraction	Structure
Wild type	Normal gravity	1.0±0.4	3.2±0.5	4.7±0.8	0.30±0.02	Flat
	Spaceflight	2.8±0.3	4.5±0.3	8.1±0.3	0.44±0.03	Column & canopy
ΔmotABCD	Normal gravity	0.4±0.1	3.9±0.4	5.6±0.7	0.28±0.03	Flat
	Spaceflight	4.2±2.4	3.7±0.1	5.2±0.4	0.28±0.05	Flat

All strains were grown in FPAs with solid inserts. Biomass and mean thickness were calculated with COMSTAT software. Void fraction was calculated as described (Equation S1). Results are shown as mean ± SD; N=3.
