# Supplementary material for: Spaceflight Promotes Biofilm Formation by Pseudomonas aeruginosa
Source: PLoS One. 2013 Apr 29;8(4):e62437. doi: 10.1371/journal.pone.0062437 (PMC3639165; doi:10.1371/journal.pone.0062437)
Supplement: Table S4 — Effects of spaceflight and motility on biofilm formation and architecture in high phosphate media. (PDF) [file pone.0062437.s008.pdf]

Table S4: Effects of spaceflight and motility on biofilm formation and architecture in high phosphate media.

In mAUM-high Pi

P. aeruginosa	**Gravity**	**Viable cells (10^6 CFU/mem)**	**Biomass ($\mu m^3/\mu m^2$)**	**Mean thickness (μm)**	**Void fraction**	**Structure**
Wild type	Normal gravity	1.0±0.7	3.2±0.1	4.3±0.5	0.24±0.08	Flat
	Spaceflight	5.5±1.5	4.4±0.2	7.5±0.6	0.40±0.05	Column & canopy
ΔmotABCD	Normal gravity	1.2±0.6	4.2±0.3	6.4±0.3	0.34±0.05	Flat
	Spaceflight	1.6±0.8	4.1±0.1	6.3±0.3	0.34±0.02	Flat

In mAUMg-high Pi

P. aeruginosa	**Gravity**	**Viable cells (10^6 CFU/mem)**	**Biomass ($\mu m^3/\mu m^2$)**	**Mean thickness (μm)**	**Void fraction**	**Structure**
Wild type	Normal gravity	0.7±0.4	3.4±0.3	4.7±1.0	0.23±0.03	Flat
	Spaceflight	8.6±2.8	5.1±0.3	8.3±1.3	0.42±0.05	Column & canopy
ΔmotABCD	Normal gravity	1.3±0.1	3.7±0.5	4.8±0.5	0.22±0.04	Flat
	Spaceflight	3.7±1.2	4.0±0.6	5.5±1.4	0.28±0.09	Flat
ΔpilB	Normal gravity	0.9±0.3	3.9±0.1	6.4±0.5	0.33±0.02	Flat
	Spaceflight	7.5±1.6	4.7±0.4	8.5±0.2	0.44±0.09	Column & canopy

All strains were grown in FPAs with solid inserts. Biomass and mean thickness were calculated from CSLM images using COMSTAT software. Void fraction was calculated as described (Equation S1). Results are shown as mean ± SD; N=3.
